# Subjective Sleepiness and Sleep Quality in Adolescents are Related to Objective and Subjective Measures of School Performance

**DOI:** 10.3389/fpsyg.2013.00038

**Published:** 2013-02-04

**Authors:** Annemarie Boschloo, Lydia Krabbendam, Sanne Dekker, Nikki Lee, Renate de Groot, Jelle Jolles

**Affiliations:** ^1^Department of Educational Neuroscience, Faculty of Psychology and Education, LEARN! Research Institute, VU University AmsterdamAmsterdam, Netherlands; ^2^Center for Learning Sciences and Technologies, Open Universiteit NederlandHeerlen, Netherlands; ^3^School for Mental Health and Neuroscience, Faculty of Health, Medicine and Life Sciences, Maastricht UniversityMaastricht, Netherlands

**Keywords:** sleep duration, sleep quality, sleepiness, school achievement, self-report, parent-report

## Abstract

This study investigated the relation between sleep and school performance in a large sample of 561 adolescents aged 11–18 years. Three subjective measures of sleep were used: sleepiness, sleep quality, and sleep duration. They were compared to three measures of school performance: objective school grades, self-reported school performance, and parent-reported school performance. Sleepiness – “*I feel sleepy during the first hours at school*” – appeared to predict both school grades and self-reported school performance. Sleep quality on the other hand – as a measure of (un)interrupted sleep and/or problems falling asleep or waking up – predicted parent-reported school performance. Self- and parent-reported school performance correlated only moderately with school grades. So it turns out that the measures used to measure either sleep or school performance impacts whether or not a relation is found. Further research on sleep and school performance should take this into account. The findings do underscore the notion that sleep in adolescence can be important for learning. They are compatible with the hypothesis that a reduced sleep quality can give rise to sleepiness in the first hours at school which results in lower school performance. This notion could have applied value in counseling adolescents and their parents in changing adolescents’ sleep behavior.

## Introduction

At the start of adolescence, major changes in sleep behavior take place. Young adolescents go to bed later than children, primarily due to a biologically driven shift in circadian rhythms. Concurrently, sleep behavior is influenced by changes in external factors, which act as “Zeitgebers”. The time at which classes start, can be an external influence forcing adolescents to wake up early on school days. The combination of late bedtimes and early rise times leads to the buildup of sleep debt during the school week (Crowley et al., 2007).

The changes in sleep behavior can have a negative effect on school performance, as shown recently in a meta-analysis by Dewald et al. (2010). Sleepiness had the strongest relationship with (inferior) school performance, followed by sleep quality and sleep duration. In their discussion, the authors state that it is unclear whether the relationship between these sleep measures and school performance depends on the indicator of school performance used. The use of objective school performance measures, such as school grades, may lead to different results than the use of self- or parent-reported school performance measures. This obscures our current understanding of relations between sleep duration, sleepiness, and sleep quality and their respective effects on performance (Dewald et al., 2010). The present study therefore investigated how subjective measures of sleepiness, sleep quality, and sleep duration relate to objective, self-, and parent-reported measures of school performance, respectively.

The major advantages of school grades as an objective measure of school performance are their high ecological validity and their reliability due to multiple measurements (Wolfson et al., 2003). However, it is often more practical and cost-effective for sleep researchers to collect data on school performance with a short questionnaire for the student or parent. Different types of self-report and parent-report questions have been used to measure school performance. Some studies used self-reported grade point average (GPA; Eliasson et al., 2002), while others asked students to indicate the level of their grades with answer options such as “mostly A’s and B’s” (Warner et al., 2008), or with a five-point scale from “far below average” to “far above average” (Maguin and Loeber, 1996).

A meta-analysis showed that self-reported grades differ only slightly from grades delivered by school administrations (Kuncel et al., 2005). When school performance is measured with a more global self-report measure such as a five-point scale, it becomes more likely that the estimation is influenced by factors such as self-esteem or peer comparison. Or, in the case of parent-reports, by how much information parents have about their child’s performance. The influence of these factors is not random: both adolescents and their parents tend to overestimate school performance (Maguin and Loeber, 1996; Kuncel et al., 2005).

Although self-reported and parent-reported school performance are not the same as school grades, they may theoretically have better construct validity than school grades (Kuncel et al., 2005). In some instances, a salient variable is more strongly related to self-reported school performance than to school grades. For example, Huang et al. (2006) found that measures of obesity were related to self-reported school performance, but not to GPA.

No studies have yet compared the relationship between sleep variables and different measures of school performance. For example, subjective “sleepiness” during the first hours at school is a totally different aspect of sleep than a student’s self-report “I have trouble falling asleep because many things cross my mind” or “I have difficulty waking up because I am deeply asleep when my mom urges me to get up.” In the present paper, three subjective measures are used, namely “sleepiness,” “sleep quality” as a measure of a more or less optimal sleep, and “sleep duration.” The study investigated the relation between these three measures and school performance in a large sample of 561 adolescents. The relationship between sleep variables and the following measures of school performance was evaluated: (1) self-reported school performance, (2) parent-reported school performance, and (3) end-of-term school grades acquired through the schools’ administrations. We hypothesized that sleepiness, sleep quality, and sleep duration would explain school performance, with sleepiness showing the largest effect size, followed by sleep quality and sleep duration. We further hypothesized that the relation between sleep variables and self- or parent-reported school performance would be different from the relation between sleep variables and school grades. The study is of potential relevance for use in school settings because of the evidence that sleep problems and inferior school performance are related. Clarification of the relation between sleep variables and school performance variables can be important in the process of counseling the student and parents as to changing the sleep behavior of the student.

## Materials and Methods

### Participants

In total, 561 adolescents participated, 243 boys and 318 girls (age *M* = 14.86 years, SD = 1.63, range = 11.83–18.95). They were in grade 7–12 of four secondary schools in the south of the Netherlands. All students followed one of the two advanced educational tracks in Dutch secondary education: higher general secondary education (42.8%) and the more difficult pre-university education (57.2%). Approximately 40% of all students in Dutch secondary education are in these two tracks (Ministry of Education, Culture, and Science, 2009). Level of parental education (LPE), the highest education level of the two parents, was between low and medium in 34.0% (at most a junior vocational education) and high in 66.0% (a senior vocational or academic education). Adolescents were excluded if they had repeated or skipped a grade after kindergarten (*n* = 89), or when data on sleep behavior or school performance were missing (*n* = 97). Participation was voluntary. Participants and their parents gave permission for participation. The Ethical Committee of VU University Amsterdam approved the research protocol.

### Procedure

The study had a cross-sectional design, and was part of a larger research project including multiple research questions. Approximately 2000 students received an information letter about the project, of whom 38% were willing to participate. Parents of participants returned a completed questionnaire on demographics and their child’s development and behavior. Participants filled in the questionnaires in the classroom, which took approximately 40 min. The questions for this study took approximately 5 min.

### Measures

#### Sleep

Sleepiness was measured by putting the following proposition to the adolescents: *I feel sleepy during the first hours at school*. Sleep quality was measured with a sum score based on four questions, which refer to distinct processes affecting sleep quality: *1. I regularly have trouble falling asleep. 2. I often wake up at night and have trouble falling asleep again. 3. I often wake up early and have trouble falling asleep again. 4. I have trouble waking up in the morning. When the alarm clock rings, I have trouble getting up*. Answers were given on a five-point Likert scale ranging from “totally agree” (five points) to “totally disagree” (one point). Sleep duration was measured by asking parents the following questions, both for school days and weekends/holidays: *What time does your child usually go to bed? What time does your child usually wake up?* Answers were in hours and minutes. Based on these bed and rise times, two sleep duration measures were calculated: time in bed (TIB) school days and TIB non-school days.

#### School performance

Objective school performance was measured with end-of-term grades (ranging from 1.0 – very bad – to 10.0 – outstanding) of the school year in which the study was carried out. The grades were acquired through the schools’ administration. School performance was measured with the mean of the subjects’ Dutch (native language), mathematics, and English as a foreign language (Reed et al., 2010). Because the schools in the sample used different grading policies, we assumed that the grades would not be comparable. Therefore, each school’s grades were transformed into *z*-scores based on the schools’ mean grade and standard deviation. Thus, academic performance was measured with the standardized mean grade for Dutch, mathematics, and English.

Self-reported subjective school performance was measured by asking adolescents the question: *How do you perform at school, compared to your classmates?* Parents answered this question about their child. Three answer options were given: “insufficient”, “average”, and “above average”.

### Analyses

To investigate the relationship between measures of sleepiness, sleep quality, sleep duration, and objective school performance, a hierarchical multiple regression analysis was performed with standardized mean grades as outcome measure. The first block consisted of the background variables age, sex, educational track, and LPE; in the second block the sleep measures were added. Similar logistic regression analyses were performed with dichotomized self-reported and parent-reported school performance. To dichotomize the scores, the “insufficient” category was dropped, because it contained less than 5% of the total sample. Thus, *n* = 537 in the logistic regression analyses with dichotomized scores consisting of the answers “average” versus “above average”. In all other analyses, *n* = 561.

## Results

Table 1 shows descriptive statistics of sleep and school performance measures. The measures of sleepiness, sleep quality, TIB on school days, and TIB on non-school days correlated between *r* = 0.00 (sleep quality and TIB on non-school days) and *r* = 0.34 (sleepiness and sleep quality, which is significant at the *p* < 0.01 level). Self-reported school performance and parent-reported school performance correlated highly (*r* = 0.66), and had good agreement, with kappa = 0.65. Both scores were *r* = 0.50 correlated to school grades, with an intraclass correlation coefficient (ICC) = 0.38 for self-reported scores and ICC = 0.40 for parent-reported scores. These are moderate effects, and similar for adolescents and parents, which indicate that adolescents and parents are equally good at estimating school performance.

**Table 1:** **Descriptive statistics of sleep and school performance**.

Variables	
Sleepiness, M (SD)	2.38 (1.13)
Sleep quality M (SD)	9.34 (3.01)
TIB school days (*hh:mm*), M (SD)	9:03 (00:40)
TIB non-school days (*hh:mm*), M (SD)	10:25 (01:01)
Standardized school grades, M (SD)	0.00 (1.00)
Self-reported school performance
Insufficient	4.3%
Average	57.4%
Above average	38.3%
Parent-reported school performance
Insufficient	4.3%
Average	53.5%
Above average	42.2%

Table 2 shows results of linear and logistic regression analyses. Significant predictors differed for the three school performance measures. School grades were predicted by sex, education track, LPE, and sleepiness. This means that girls, students from pre-university education, and students with highly educated parents achieved the highest school grades. In addition, sleepy students achieved lower grades than their peers. Sleep quality and sleep duration were not related to school grades.

**Table 2:** **Relations between sleep and school performance measures: results of linear and logistic regression analyses**.

Predictors	Standardized mean grades^a^	Self-reported school performance^b^	Parent-reported school performance^b^
	Beta	Exp(B)	Exp(B)
Block 1	*R*^2^ = 0.12	Cox & Snell *R*^2^ = 0.01	Cox & Snell *R*^2^ = 0.06
Age	−0.03	0.98	1.03
Sex	0.15**	1.20	1.66**
Education track	0.28**	1.42	2.36**
LPE	0.09*	1.26	1.12
Block 2	*R*^2^ = 0.13	Cox & Snell *R*^2^ = 0.06)	Cox & Snell *R*^2^ = 0.09
Age	−0.04	1.06	1.08
Sex	0.16**	1.31	1.86**
Education track	0.27**	1.42	2.43**
LPE	0.08*	1.21	1.06
Sleepiness	−0.12**	0.77**	0.92
Sleep quality	−0.02	0.95	0.91**
TIB school days	−0.03	1.35	1.31
TIB non-school days	−0.05	0.84	0.84

*LPE, level of parental education; TIB, time in bed*.

*^a^Linear regression analyses, *N* = 561; ^b^logistic regression analyses, *N* = 537*.

*p < 0.05, **p < 0.01.

Self-reported school performance was significantly related to sleepiness only, with sleepy students reporting lower performance than their peers. None of the associations with background variables or other sleep variables were significant. Parent-reported school performance was predicted by sex, education track, and sleep quality. According to parents, girls, students from pre-university education, and those with good sleep quality had higher school performance than their peers.

## Discussion

The current study investigates several subjective measures of sleep, and both objective and subjective measures of school performance in adolescents. The study was set up to evaluate the hypothesis that the measures used might determine whether or not a relation is found between sleep and school performance. The findings show that this is indeed the case; the implication is therefore that further research on sleep and school performance should take the important question “what do we measure and what does it refer to?” into account. The measure “sleepiness” gives an indication of reduced alertness and attention during the first hours at school. This measure was used because of the fact that many adolescents have complaints of distractedness and attentional problems especially in the first hours at school. Indications exist that such problems and subjective sleepiness might be related to more or less disturbed sleep. Research has shown that a shift occurs in the 24 h diurnal rhythm in young adolescents. This can have as a result that the student goes to bed at a later moment than in his childhood. And this causes a difficulty in getting up. The student may in fact have a shorter night than is optimal for proper functioning during the school day: the restorative function of sleep might therefore be compromised. The findings in this study indeed show that sleepiness in the first hours at school is correlated both to lower school grades and to lower self-reported school performance. A different finding was done for the second sleep measure, namely “sleep quality.” This measure gives an indication that the student has difficulty falling asleep, has trouble waking up and/or wakes up at night, and has trouble falling asleep again. This second measure appeared to be related to parent-reported (but not student-reported) school performance. No relation was found to school grades. The third measure (the duration of the sleep as reported by parents) was not related to any of the school performance measures. Therefore, the fact that previous studies did not always find expected relations between sleep and school performance (see Dewald et al., 2010) may be due to the use of different school performance measures. In future studies, it is of major importance to carefully choose the relevant measure. The findings done in the present study underscore the notion that sleep is important for performance at school in adolescents. An implication is that school performance could be improved by creating the conditions in which the students is less sleepy, more alert, and also has a better sleep quality. As the present study was set up as a large scale epidemiological study, future studies must go in depth with respect to more carefully defining and measuring sleepiness and sleep quality, and also evaluating individual students at more time points over the day and even through the week. This type of studies is presently underway.

With respect to the measurement of subjective school performance reported by adolescents and by parents, a methodological remark has to be made. The student and the parent-reports seemed quite similar at first sight: they were both moderately good in estimating school performance. However, self- and parent-reported school performances do appear to measure different concepts: parent-reported school performance was related to sex and educational track, while student-reported school performance was not. This must be due to differences between students and their parents in the evaluation of school performance and in the comparison group used. Adolescents probably compare themselves with peers who are very similar to them, are of the same sex, and from the same educational track. That is their frame of reference. Besides, the students will have a different opinion as to what constitutes “appropriate school performance” in comparison to their parents. The parents can be expected to evaluate the performance of their children with a broader reference group and against more abstract norms. Future research should clarify this issue by acquiring more information about the reference group used by adolescents and parents and on the attitudes with respect to what they consider as appropriate school performance.

The current study illustrates that self-reported school performance, parent-reported school performance, and school grades do not measure the same construct. To understand more of the complex relations between the various sleep measures and their effects on school performance, differences between school performance measures should be included in future studies. Accordingly, they should choose a school performance measure that best fits their research question. Furthermore, to avoid confusion, it would be helpful to mention explicitly which measure is used, for example by speaking about “self-reported school performance” or “objective school performance”.

There are implications of the study for school practice and for education in a broad sense. The finding that sleepiness correlates to performance can be used in counseling the student and his or her parents. A first step is to acknowledge the fact that sleepiness can be due to problems falling asleep or waking up or other aspects of sleep behavior. The student and his or her parents can acquire insight as to the factors which improve the risk for sleepiness. This gives the student more control over his sleep behavior and help him to seek guidance or “tips” in creating the conditions which lead to better sleep and decreasing fatigue at school. More attention for the subjects taught at school could lead to better school grades and also more motivation to learn and perform. All in all: it can show both student, teacher and parents that performance at school is the result of more than only didactical procedures, and that biological factors can also be of major importance … and that the student and his or her parents can learn to create the conditions at home which have positive impact on functioning at school.

## Conflict of Interest Statement

The authors declare that the research was conducted in the absence of any commercial or financial relationships that could be construed as a potential conflict of interest.
